# Locus Coeruleus magnetic resonance imaging in cognitively intact elderly subjects

**DOI:** 10.1007/s11682-021-00562-0

**Published:** 2021-11-05

**Authors:** Filippo Sean Giorgi, Francesco Lombardo, Alessandro Galgani, Hana Hlavata, Daniele Della Latta, Nicola Martini, Nicola Pavese, Irene Ghicopulos, Filippo Baldacci, Alessio Coi, Marco Scalese, Luca Bastiani, Petra Keilberg, Daniele De Marchi, Francesco Fornai, Ubaldo Bonuccelli

**Affiliations:** 1 Neurology Unit, Pisa University Hospital, Pisa, Italy; 2 Department of Translational Research and of New Surgical and Medical Technologies, University of Pisa, Pisa, Italy; 3 Cardiovascular and Neuroradiological Multimodal Imaging Unit, Fondazione “G. Monasterio”, National Research Council/Tuscany Region, Pisa, Italy; 4 Deep Health Unit, Fondazione “G. Monasterio”, National Research Council/Tuscany Region, Pisa, Italy; 5 Clinical Ageing Research Unit, Newcastle University, Newcastle upon Tyne, UK; 6 Institute of clinical Medicine, PET Centre, Aarhus University, Aarhus, Denmark; 7 Institute of Clinical Physiology of National Research Council, Pisa, Italy; 8 IRCCS Neuromed, Pozzilli, Italy

**Keywords:** Locus Coeruleus, Neuromelanin, MRI, Normal ageing, Neuropsychology

## Abstract

The locus coeruleus is the main noradrenergic nucleus of the brain and is often affected in neurodegenerative diseases. Recently, magnetic resonance imaging with specific T1-weighted sequences for neuromelanin has been used to evaluate locus coeruleus integrity in patients with these conditions. In some of these studies, abnormalities in locus coeruleus signal have also been found in healthy controls and related to ageing. However, this would be at variance with recent post-mortem studies showing that the nucleus is not affected during normal ageing. The present study aimed at evaluating locus coeruleus features in a well-defined cohort of cognitively healthy subjects who remained cognitively intact on a one-year follow-up. An ad-hoc semiautomatic analysis of locus coeruleus magnetic resonance was applied. Sixty-two cognitively intact subjects aged 60–80 years, without significant comorbidities, underwent 3 T magnetic resonance with specific sequences for locus coeruleus. A semi-automatic tool was used to estimate the number of voxels belonging to locus coeruleus and its intensity was obtained for each subject. Each subject underwent extensive neuropsychological testing at baseline and 12 months after magnetic resonance scan. Based on neuropsychological testing 53 subjects were cognitively normal at baseline and follow up. No significant age-related differences in locus coeruleus parameters were found in this cohort. In line with recent *post-mortem* studies, our in vivo *study confirms* that locus coeruleus magnetic resonance features are not statistically significantly affected by age between 60 and 80 years, the age range usually evaluated in studies on neurodegenerative diseases. A significant alteration of locus coeruleus features in a cognitively intact elderly subject might be an early sign of pathology.

**Supplementary Information:**

The online version contains supplementary material available at 10.1007/s11682-021-00562-0.

## Introduction

The locus coeruleus (LC) is the main noradrenergic (NE) nucleus of the brain. It is located in the pons, below the floor of the fourth ventricle, extending along the rostro-caudal axis for up to approximately 16 mm (Paxinos & Mai, 2003). LC neurons densely innervate cortical and subcortical structures, and the nucleus plays a key role in wake/sleep cycle regulation and several cognitive functions (Aston-Jones & Cohen, 2005; Berridge et al., 1993; Berridge & Waterhouse, 2003; Sara, 2009). The LC degenerates in Parkinson’s disease (PD) and Alzheimer’s disease (AD) (Braak et al., 2003, 2011; Gesi et al., 2000; Kelly et al., 2017). Conversely, a recent detailed post-mortem study in a large cohort of subjects has shown that LC does not degenerate during normal ageing (Theofilas et al., 2017), confirming previous smaller studies (Fernandes et al., 2012; Mouton et al., 1994; Ohm et al., 1997), but challenging a previous post-mortem observation (Manaye et al., 1995).

Recent advancements in Magnetic Resonance Imaging (MRI) technology have enabled the assessment of the LC in vivo. LC neurons contain neuromelanin, a by-product of NE catabolism which is a chelator of metal ions (Martin-Bastida et al., 2017). The combination of neuromelanin with ions and macromolecules (including lipids and proteins) within the LC likely contributes to T1-shortening effects and can be visualized on T1-weighted images (Betts et al., 2019b). Over the past 15 years, a number of LC-MRI studies have been performed in patients affected by neurodegenerative diseases with different imaging protocols, including 2D-Fast Spin Echo (FSE)-T1, or Inversion Recovery-weighted images, or Magnetization Transfer (MT) approach, using 2D Gradient Echo (GRE) or 3D prepared spoiled GRE (SPGR) or Turbo FLASH (TFL) sequences, in 1,5 T, 3 T and 7 T scanners (Chen et al., 2014; Dahl et al., 2019; García-Lorenzo et al., 2013; Keren et al., 2015; Liu et al., 2017, 2019, 2020; Nakane et al., 2008; Priovoulos et al., 2018; Sasaki et al., 2006; Schwarz et al., 2017; Shibata et al., 2006). An issue with these studies is that different approaches of LC-MRI acquisition and analysis have been used, and only very recently a more standardized methodological framework for LC imaging analysis has been suggested (Betts et al., 2019a, b). Additionally, some authors have also reported LC abnormalities in healthy elderly subjects (Betts et al., 2017; Dahl et al., 2019; Liu et al., 2019; Shibata et al., 2006), suggesting an age-related alteration of LC-MRI signal, either involving the whole nucleus (Shibata et al., 2006), or limited to its rostral part (Betts et al., 2017; Betts et al., 2019a, b; Liu et al., 2019). These findings in healthy subjects appear to be at variance with the most recent *post-mortem* findings mentioned above (Theofilas et al., 2017) but could also be related to a very initial sublinical pathology in these subjects.

Getting a better understanding of age-related features of LC-MRI in healthy controls is important for interpreting LC-MRI data obtained in age-matched patients with neurodegenerative diseases. Therefore, in the present study, a population of healthy aged subjects was prospectively evaluated over a one-year period. An extensive neuropsychological analysis was performed both at baseline, to rule out the occurrence of any cognitive impairment, and after prolonged follow-up, to confirm cognitive integrity. A semi-automated method of analysis of LC-MRI scans was applied to asses LC-contrast ratio, as performed in other studies, but also the number of voxels likely belonging to the LC for a better evaluation of LC integrity.

## Methods

The study was conducted at the Pisa University-Hospital Neurology Clinic and the MRI scans were performed at Fondazione “G.Monasterio”-CNR/Tuscany Region. Participants were recruited among volunteers who learned about the study from one of the researchers involved. The study protocol was approved by the Ethics Committee of Tuscany Region Area Vasta Nord-Ovest; all included subjects provided written informed consent.

### Subjects

Participants were males/females, aged 60 to 80 years with no subjective and/or objective complaint of cognitive impairment and completely autonomous in their daily routine (Jack et al., 2018). Exclusion criteria were: severe medical/cardiological comorbidities; psychiatric comorbidities; neurological disease potentially associated with cognitive decline; history of drugs/alcohol abuse; contraindications to MRI; MRI signs of moderate-severe chronic vascular encephalopathy, according to (Fazekas et al., 1987), or other significant alterations.

### Neurological and neuropsychological evaluation

All participants were submitted to a pre-screening analysis by receiving Mini-Mental State Examination (MMSE) (Magni et al., 1996). Subjects with MMSE score > 24/30, underwent complete neuropsychological examination (T0 assessment), including Digit Span (Orsini et al., 1987) and Corsi Block Span (Orsini et al., 1987) for working memory, Rey Auditory Verbal Learning Test (Carlesimo et al., 1996) (RAVLT), Story Learning Test (Carlesimo et al., 2002) and Free and Cue Selective Reminding Test (Sarazin et al., 2007) for verbal memory, Rey–Osterrieth complex figure (Carlesimo et al., 2002) for visuo-spatial memory, Trail Making Test A,B and B-A (Giovagnoli et al., 1996), Stroop test (Caffarra et al., 2002), Attentional Matrices (Spinnler & Tognoni, 1987), and Digit symbol substitution (Lang et al., 2013), Clock Test (Royall et al., 1998) and Phonemic Fluency Test (Carlesimo et al., 1996) for executive, attentive and praxis functions (see Table 1 and 2 for sub-sections of each test). Test scores were standardized for age and educational level, according to the normative studies cited above.

**Table 1:** Neuropsychological assessment

Age	71.70 + 4.69 y	T0		T1		LMM		*p*
M: F	20: 33	Mean	SD	Mean	SD	NR		
Educational level (years)		9.45	4.65	–	–			
Mini Mental State Examination		27.04	1.27	27.33	1.20	>24	0.29 (−0.11/0.7)	0.356
Working Memory	Verbal- Digit span	5.47	0.67	5.50	0.72	>3.75	0.03 (−0.2/0.25)	0.797
	Visual- Corsi Blocks test	4.57	0.56	4.61	0.55	>3.5	0.04 (−0.16/0.25)	0.747
Verbal Memory	Rey Auditory Verbal Learning Test – Immediate Recall	42.32	8.18	45.16	8.36	>28.53	2.84 (0.15/5.53)	0.390
	Rey Auditory Verbal Learning Test – Delayed Recall	8.54	2.60	9.32	2.42	>4.69	0.77 (−0.07/1.62)	0.237
	Story – Immediate Recall	6.32	1.79	6.77	1.46	>3.10	0.46 (−0.11/1.02)	0.283
	Story – Delayed Recall	6.09	2.13	7.05	1.37	>2.39	0.96 (0.38/1.54)*	0.020
	Free and Cue Selective Reminding Test	31.08	6.71	31.40	6.20	>16	0.32 (−1.89/2.53)	0.818
Visual Memory	Rey–Osterrieth complex figure – Copy	29.75	5.36	30.83	5.00	>23.76	1.07 (−0.68/2.82)	0.464
	Rey–Osterrieth complex figure – Immediate Recall	16.65	6.13	18.73	6.65	>6.44	2.08 (0.01/4.15)	0.327
	Rey–Osterrieth complex figure – Delayed Recall	16.14	5.73	17.99	5.93	>6.33	1.86 (−0.13/3.84)	0.264
Attention. Executive and Praxis functions	Attentional Matrices	50.61	7.04	51.59	6.61	>31	0.98 (−1.23/3.2)	0.642
	Digit symbol substitution	13.23	2.76	13.66	2.87	>5	0.43 (−0.47/1.34)	0.633
	Stroop Color-Word Test – time interference effect	13.42	12.90	11.45	12.47	<36.91	−1.97 (−6.5/2.56)	0.608
	Stroop Color-Word Test – error rate	0.90	1.45	0.49	1.06	<4.23	−0.41 (−0.88/0.06)	0.249
	Trail Making Test A	55.69	35.39	47.35	24.65	<94	−8.33 (−16.83/0.16)	0.270
	Trail Making Test B	95.55	81.94	85.85	76.95	<283	−9.7 (−34.1/14.7)	0.623
	Trail Making Test B-A	47.05	69.69	42.06	60.41	<187	−4.99 (−26.12/16.14)	0.758
	Clock Test	12.70	1.60	12.89	1.50	>10	0.19 (−0.31/0.69)	0.615
	Phonemic Fluency Test	36.35	9.48	37.20	9.29	>17.35	0.85 (−2.35/4.04)	0.753

NR: normality range; T0: evaluation at baseline; T1: evaluation after 12-months follow-up; LMM: linear mixed model coefficient (95% CI); * statistically significant

**Table 2:** Correlations between LC-MRI parameters and neuropsychological tests

		T0				T1			
		Vox		LC-CR		Vox		LC-CR	
		r	p	r	p	r	p	r	p
Educational level Mini Mental State Examination		0.105	*0.456*	0.182	*0.193*	–	*–*	–	–
		−0.216	1.000	0.049	0.320	0.067	0.790	0.147	0.651
Working memory	Verbal- Digit span	0.011	0.988	0.087	0.340	0.112	1.000	0.104	0.918
	Visual- Corsi Block Test	−0.178	1.000	0.017	1.000	0.095	0.992	0.175	0.600
Verbal Memory	Rey Auditory Verbal Learning Test – Immediate Recall	−0.109	0.969	−0.07	0.925	0.132	1.000	0.07	0.828
	Rey Auditory Verbal Learning Test – Delayed Recall	−0.019	0.993	−0.331	0.744	−0.009	0.952	0.052	0.840
	Story – Immediate Recall	0.042	1.000	0.015	0.853	−0.078	0.962	0.093	0.785
	Story – Delayed Recall	−0.029	1.000	−0.098	1.000	−0.072	0.867	0.086	0.771
	Free and Cue Selective Reminding	−0.105	0.908	−0.118	1.000	0.025	0.906	0.098	0.885
Visual Memory	Rey–Osterrieth complex figure – Copy	−0.025	1.000	0.187	1.000	0.152	1.000	0.217	0.793
	Rey–Osterrieth complex figure – Immediate Recall	−0.003	0.982	0.031	1.000	0.05	0.847	0.293	0.660
	Rey–Osterrieth complex figure – Delayed Recall	−0.049	1.000	0.044	1.000	0.107	1.000	0.199	0.770
Attention. Executive and Praxis functions	Attentional Matrices	−0.164	0.800	0.053	1.000	0.047	0.819	−0.003	1.000
	Digit symbol substitution	−0.177	1.000	0.184	1.000	0.103	1.000	0.197	0.628
	Stroop Color-Word Test – time interference effect	0.088	0.883	−0.083	1.000	−0.232	1.000	0.043	0.844
	Stroop Color-Word Test – error rate	0.169	0.900	−0.029	1.000	−0.072	0.813	0.094	0.840
	Trail Making Test A	0.091	0.938	−0.159	1.000	−0.093	0.924	0.003	0.986
	Trail Making Test B	0.087	0.828	−0.08	0.971	−0.153	1.000	−0.158	0.645
	Trail Making Test B-A	0.129	0.890	0.035	0.931	−0.075	0.915	−0.197	0.527
	Clock Test	0.14	0.906	0.185	0.954	0.096	1.000	0.229	0.990
	Phonemic Fluency Test	−0.194	1.000	0.291	0.917	0.11	1.000	0.069	0.781

r: Pearson’s coefficient; T0: evaluation at baseline; T1: evaluation after 12-months follow-up;

Only subjects with a neuropsychological profile within normal ranges according to the above-quoted normative studies were finally included in the study. Neuropsychological evaluation and MRI scan were performed within 30 days of each other. Subjects were re-evaluated clinically/neuropsychologically with the same battery of tests at 12 + 1 months (T1).

### MRI protocol

All MR data were acquired with a 3.0-Tesla MR-Unit (GE Excite HDx, GE, USA) using an 8-channels phased-array head coil. In addition to LC-sensitive sequence, images of the entire brain were also acquired. The protocol included routine 2D-FLAIR (Fluid Attenuation Inversion Recovery), T2* GRE, Spin Echo(SE) T1- and FSE T2-weighted with fat saturation and diffusion-weighted imaging.

###  Locus Coeruleus-sensitive sequence and post-processing analysis

For LC imaging, the LC-sensitive sequence used was 2D-FSE T1-weighted, acquired along the oblique axial plane, perpendicular to the fourth ventricle floor, covering an area from the pons inferior border to the posterior commissure: TR 600 ms; TE 14 ms; flip angle 90°; echo train length 2; number of excitations (NEX) 5; matrix size 512 × 384; FOV 200 × 200 mm; pixel size 0.39 × 0.52 mm; 12 contiguous slices, slice thickness 2.2 mm, slice gap 0; acquisition time 14.29 min. This rostro-caudal region was selected to make sure including the whole LC in all of the subjects (Fernandes et al., 2012).

**Fig. 1:**
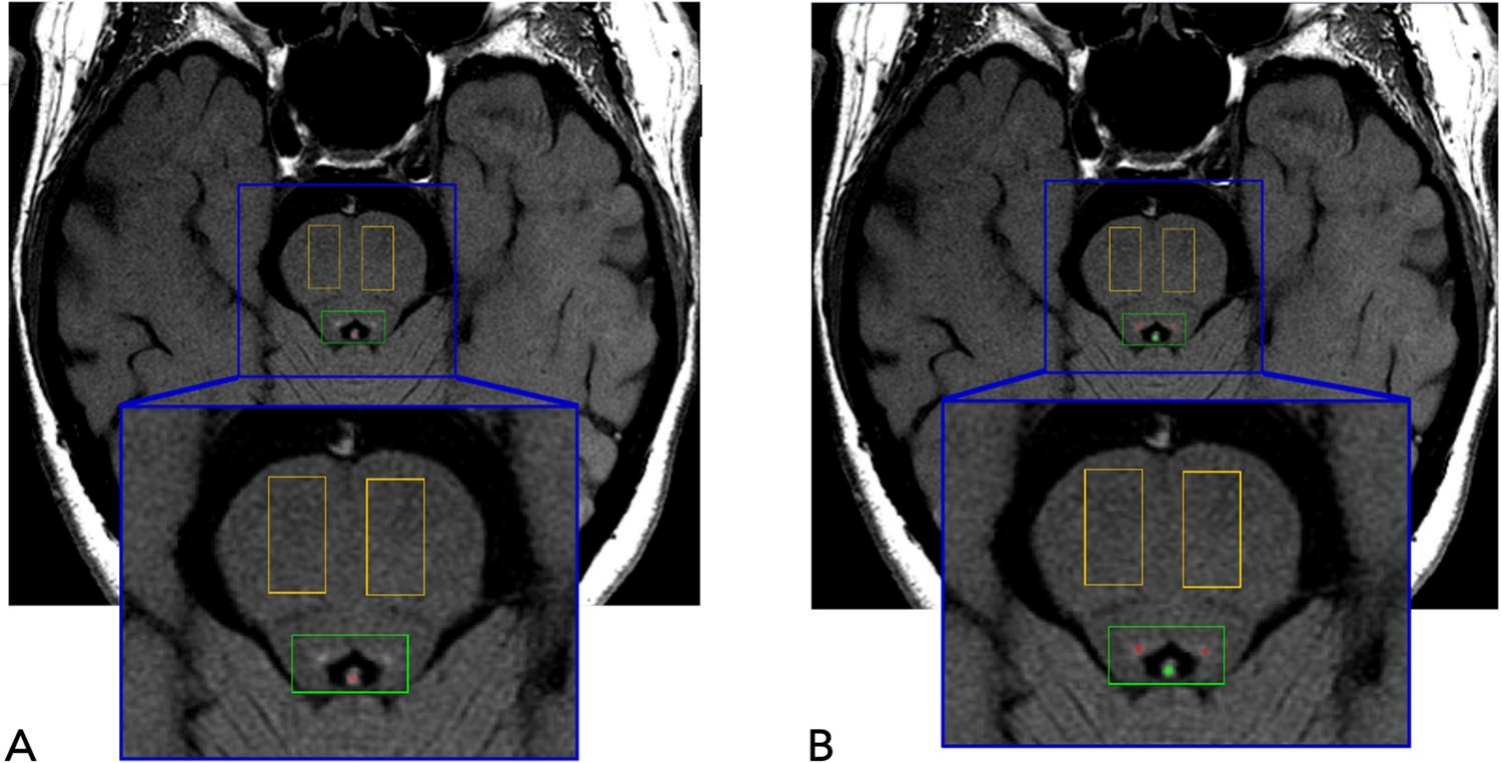
Method of LC identification in T1-weighted neuromelanin-sensitive LC images. The figure provides an exemplification of the sequential steps of LC analysis. Panels **A** and **B** show a representative single slice. Bilateral reference ROIs in the ventral pons, used to normalize the LC-signal intensity, are shown in orange; the ROI including the two LC nuclei is shown in green. **A.** First, the software automatically highlights in red the 10-connected voxels within the LC ROI (in consecutive slices-not shown) with the brightest intensity for each side. Then, the operator recognizes the voxels as anatomically incongruent for LC (e.g. artifacts in the fourth ventricle) and deselect them (marked as green). **B.** The threshold is then updated by the software and new voxels with the highest intensity are highlighted in red

A semi-automatic procedure was used to calculate LC signal intensity and the estimated number of voxels belonging to LC, profiting from a software developed in-house in Java language. Measurements were carried out by two trained operators and an analysis of intra- and inter-rater variability was performed (see Results). The workflow analysis was composed of sequential steps performed in the LC-sensitive images (Fig. 1). The operator placed three rectangular regions of interest (ROIs, 78 mm^2^ each, same size/shape) in the first slice containing the LC-complex: two were placed bilaterally in the ventral pons and used as reference to normalize the signal intensity of LC, the third was placed at the level of the fourth ventricle floor to include the LC/subcoeruleus complex bilaterally; ROIs placement in the first slice was automatically maintained in the remaining contiguous slices. At this stage, the software automatically calculated the intensity threshold, in accordance with García-Lorenzo et al. (García-Lorenzo et al., 2013); specifically, the 10-connected voxels with the maximum intensity were highlighted in both the LC-ROIs, and the threshold was obtained as the lowest signal intensity of these voxels. Then, the software selected the initial set of candidate voxels of the LC as the ones with intensity values higher than the extrapolated threshold. At this stage, if the operators recognized that highlighted voxels were anatomically incongruent with LC (e.g. fourth ventricle artifacts), they marked them as excluded. The threshold was then updated and all voxels in LC-ROI above that threshold were highlighted and from these candidate LC voxels the operators deselected the ones that they judged as not anatomically consistent with LC (likely due to signal noise). In particular, the operators excluded voxels that were located close to the midline or separated from the main cluster of voxels selected by the software. From the remaining voxels two parameters were obtained: a) the mean LC-Contrast Ratio (LC-CR) of the voxel positive for LC signal, i.e. the ratio between the mean signal intensity measured in the LC region (LC_intensity_) and the mean signal intensity of the reference regions (PT_intensity_) **[LC-CR = (LC**_**intensity**_**/PT**_**intensity**_**]**, denoted as “LC-CR” parameter; b) the estimated number of voxels belonging to LC (“Vox” parameter) computed as the total number of LC voxels surviving the above-mentioned analysis. For each subject, the final value considered for each parameter was the result of the mean of the four values, obtained by two measurements by each one of the two operators.

### Statistical analysis

Neuropsychological evaluations at baseline and follow-up were compared using multilevel mixed-effects linear regression. To assess associations between Vox, LC-CR, age, and neuropsychological tests, Pearson correlation coefficient was used. A two-sided p < 0.05 was considered statistically significant. When multiple hypotheses were tested false discovery rate (FDR) correction was applied. Inter- and intra-rater agreement of measurements were based on coefficient of variation (CV) and intraclass correlation coefficients (ICC) in all subjects. The ICC’s were computed as per (Shrout & Fleiss, 1979) based on the two-way mixed effect analysis of variance model with the absolute agreement type being selected for ICC calculations. The Inter- and Intra-CV were calculated like an average value calculated from the individual CVs for all the duplicate measurements. Inter-CVs of less than 15% are considered generally acceptable, while Intra-CVs should be less than 10% (Guidance for industry: bioanalytical method validation, 2001). Statistical analyses were performed using STATA software [Stata Statistical Software: R13. StataCorp LP].

## Results

Sixty-two subjects without any subjective cognitive complaint were recruited. However, nine were excluded from the final analysis for low MMSE, or due to MRI movement artifacts or because cognitively impaired in the neuropsychological tests at baseline or at follow-up. Thus, 53 subjects were included in the final analysis. The mean age at MRI was 71.70 + 4.69, male/female ratio 20/33. The mean educational level was 9.45 + 4.65 years (range 5–17). Neuropsychological battery results were within normality ranges for all of the subjects included in the final analysis, both at baseline and at follow-up (Table 1). Multilevel mixed-effects linear regression, FDR-corrected for multiple comparisons, showed no relevant differences in the results of neuropsychological tests by time (Table 1) with the exception of the delayed recall of the Story Learning Test (ρ^2^: 0.96 [0.38/1.54]; p = 0.020), which showed a positive effect of time. However, the scores of each test for each one of the included participants remained always within the normality range.

### LC-MRI results and intra−/inter-rater agreement

Intra- and inter-observer agreements were assessed by calculating both the ICC and CV. There was an intra- and inter-observer agreement for both LC-CR and Vox (Supplementary Table 1). The mean Vox value was 13.62+ 3.58 (median: 14.50), while mean LC-CR value was 1.265 + 0.037 (median: 1.262). There was no significant correlation between Vox and LC-CR (r: 0.088; p = 0.529) (Supplementary Fig. 1).

### Correlation of Locus Coeruleus parameters with age

**Fig. 2:**
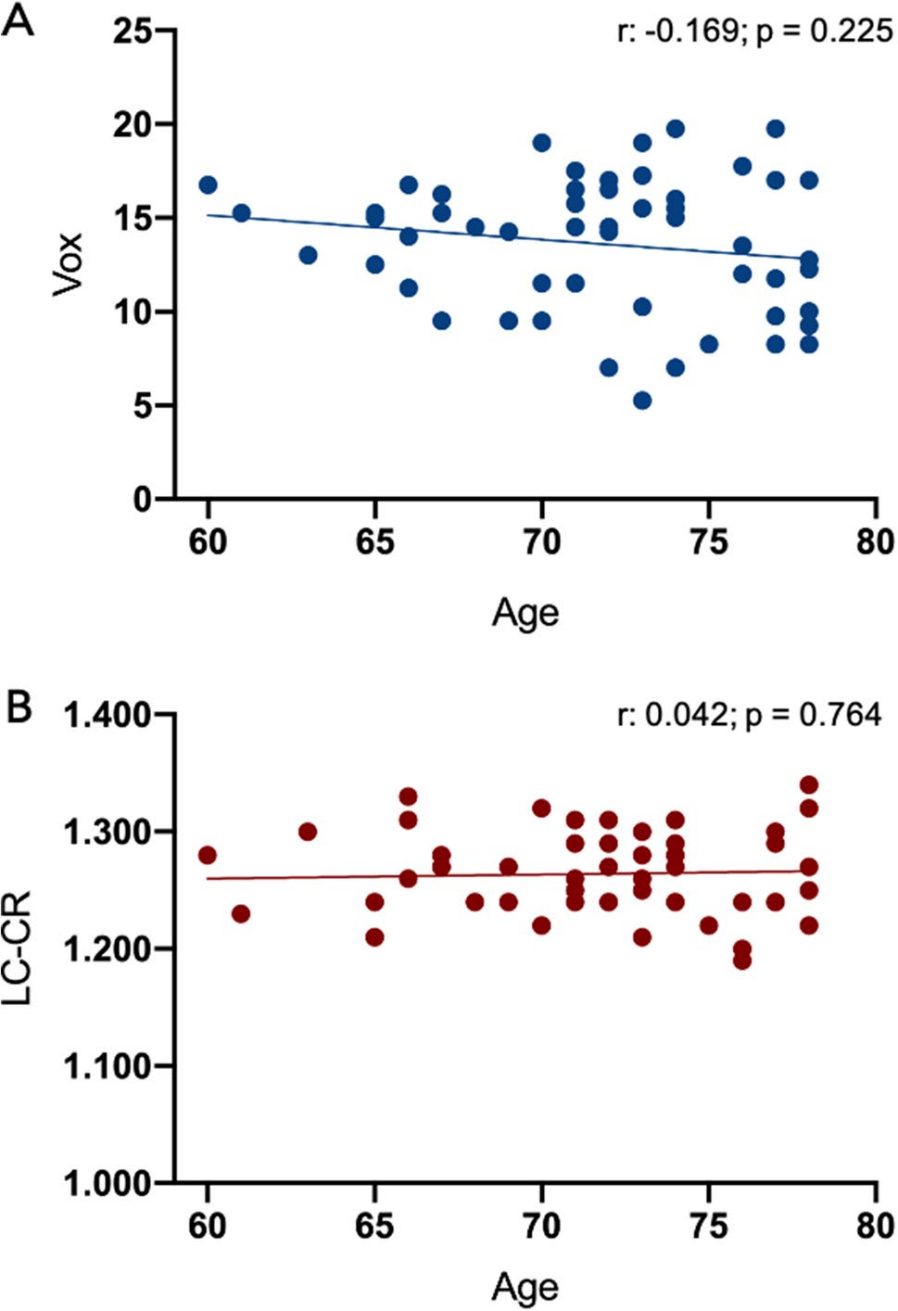
LC-MRI data in cognitively healthy subjects. Distribution of the volumetric parameter Vox (graph **A**) and intensity parameter LC-CR (graph **B**) by age: no significant relationship between these two parameters and ageing could be observed. r: Pearson’s coefficient

The correlations of the two MRI-LC parameters with age for the included subjects were not significant (Vox, r: −0.169; *p* = 0.225; LC-CR, r: 0.042; *p* = 0.764) (Fig. 2).

### Relation between Locus Coeruleus parameters with neuropsychological tests

Linear correlation analysis did not show any relevant correlation between the single subject’s LC-MRI parameters and neither the MMSE score (Supplementary Fig. 2), nor the results of neuropsychological tests in the included subjects, both at baseline and follow-up (Table 2).

### Analyses in excluded participants

In the three subjects excluded for cognitive impairment, Vox was 9.1, 7.5 and 7.3, respectively, and LC-CR was 1.261, 1.227 and 1.250, respectively, and in the three subjects excluded for low baseline MMSE, Vox was 17.75, 16.75 and 15.00, respectively, and LC-CR was 1.257, 1.289 and 1.266, respectively.

## Discussion

In the present study, the mean intensity and the number of voxels compatible with LC were assessed in cognitively intact subjects aged 60–80 years by LC-related MRI sequences. We found that both LC parameters (LC-CR and Vox) were not significantly correlated with age in this group. Lack of cognitive alteration was confirmed at follow-up by extensive neuropsychological testing.

Neuromelanin is a catabolic by-product of catecholamines whose accumulation within LC neurons increases during ageing reaching a *plateau* around 60 years of age (Zecca et al., 2004). Recently, it has been shown *post-mortem* by stereological analysis, that the number of LC neurons does not vary significantly during normal ageing (Theofilas et al., 2017) confirming similar previous studies (Fernandes et al., 2012; Mouton et al., 1994; Ohm et al., 1997) but challenging an older one (Manaye et al., 1995) and others quoted in Manaye et al. (1995). The latter, however, were performed without stereological analysis and in relatively small samples of subjects. Conversely, a marked LC degeneration has been repeatedly confirmed in Parkinson’s Disease (PD) (Gesi et al., 2000) and AD (Kelly et al., 2017).

In the last decades, different approaches for estimating LC features by LC-related MRI have been used ranging from fully manual to semi-automated segmentation, in native space or template (Betts et al., 2017; Chen et al., 2014; Dahl et al., 2019; García-Lorenzo et al., 2013; Liu et al., 2019; Shibata et al., 2006) (see also the review by Liu and colleagues (Liu et al., 2017) and the studies mentioned in the consensus by Betts et al. (2019b). We developed a semi-automatic segmentation method partially inspired by the one used by García-Lorenzo and colleagues (García-Lorenzo et al., 2013) with a couple of significant differences. Firstly, in the present study, the reference ROIs and the LC-ROI were defined directly on the native LC-sensitive images, rather than on template. Secondly, since the operator could deselect a variable number of LC voxels in the LC-ROI from those automatically selected by the software (making this method semi-automated), this analysis method also provided an estimation of the number of voxels belonging to the LC. The analysis of the LC-CR and LC-Vox (i.e. number of voxels belonging to LC) was performed directly in the native space although we are aware that recent works (Betts et al., 2017; Dahl et al., 2019; Liu et al., 2019; Liu et al., 2020) have applied a sophisticated analysis technique profiting of a template-based approach. Using the template for the LC segmentation mask could lead to a potential bias in volume estimation in individual subjects (Betts et al., 2017; Dahl et al., 2019; Liu et al., 2019, 2020). Thus, we chose to perform LC analysis in the native space with the main aim of calculating the number of voxels belonging to LC in the single subjects. Furthermore, working in native space eliminates the need for interpolation into a template space, which might per se lead to image blurring. It is worth noting that estimation of LC voxel number has been performed in only 6 subjects by Chen et al. (Chen et al., 2014) using analysis in the native space.

That being said, the LC intensity assessed by our method in the present group of subjects did not show a statistically significant correlation with age, confirming ex-vivo evidence (Fernandes et al., 2012; Mouton et al., 1994; Ohm et al., 1997; Theofilas et al., 2017). Previous data proposing an age-related LC neuron loss (German et al., 1988; Manaye et al., 1995) have been questioned because of potential biases due to morphological analysis and subjects’ selection. Among LC-MRI studies, Shibata et al. (Shibata et al., 2006) showed that LC signal increases in younger subjects, reaching a *plateau* at around fifty years, and then it declines in the age group between 60 and 70 years. Clewett et al. (Clewett et al., 2016), and Betts et al. (Betts et al., 2017) compared MRI LC-CR from old subjects with those of young ones, and showed a higher LC-CR in the former group, but they did not specifically assess LC correlation with age. Recently, in a very large sample of healthy subjects Liu et al. (Liu et al., 2019) found an increase of whole LC-CR up to 57 years, which remained stable in the older subgroup. In the study by Liu et al. (2019) subregional analysis revealed a higher LC-CR in the rostral part compared with the caudal region of the LC in aged group, and rostral LC-CR showed an inverted-U relationship with age. Dahl et al. (Dahl et al., 2019) did not show reliable differences in mean LC-CR between young and older adults, but observed a trend towards a decrease of LC-CR in rostral segments in the latter group. The present results concerning LC-CR are in line with the whole-LC-CR results by Liu et al. (Liu et al., 2019) and Dahl et al. (Dahl et al., 2019), concerning the age group between 60 and 80 years, while we did not extrapolate sub-regional informations from our group of subjects. In fact, the present post-processing protocol on native space was not designed to allow delineating a rostral-vs caudal part of LC, due to intrinsic limitations in terms of anatomical detailing of LC features. This might represent a limit of our approach compared with those by (Dahl et al., 2019; Liu et al., 2019).

As detailed above, the semiautomatic method used in the present study provides an estimated number of voxels belonging to LC, which might represent an indirect index of LC volume (with the abovementioned limits of making anatomical correlation of LC-MRI data). This parameter was characterized by a certain degree of variability within our population, despite the quite narrow age range considered, which is in line with post-mortem studies (Fernandes et al., 2012; Theofilas et al., 2017). However, no statistically significant correlation between subjects’ age and Vox was found, although a slight negative trend could be observed (Fig. 2). Such a trend could be explained in light of two considerations. First, it could not be excluded that some of the older ones from the present group of subjects may develop cognitive impairment in the future, although their neuropsychological assessment was preserved at the 12-months follow-up. Second, ex-vivo stereological studies demonstrated the occurrence of LC shrinkage through aging, in the absence of concomitant neuronal loss (Theofilas et al., 2017); this phenomenon could partly contribute to the slight trend we observed through indirect LC markers. Thus, LC-Vox may represent an easily measurable parameter in studies comparing healthy individuals and patients with neurodegenerative diseases enrolled in clinical trials.

In our sample, we did not find any correlation between LC-CR and Vox. This means that subjects with different Vox number may have a similar mean LC-CR and vice-versa. Thus, a smaller LC, as estimated by Vox, is not necessarily associated to a lower mean CR. We cannot further speculate on this lack of correlation, as the precise meaning of CR is not fully known yet (as it may reflect the neuromelanin concentrations, the neuronal density, or both, and other factors as well-Betts et al., 2019a, b). The lack of correlation might be also related, at least in part, to the fact that in the present study only cognitively intact subjects were studied, and/or to the relatively low number of subjects assessed. This correlation is worth being addressed by further studies. Nevertheless, the fact that both parameters, although unrelated with each other, do not show a relation with age may further strengthen the interpretation of a lack of age-related effect on LC degeneration in normal elderly.

The main reason for the detailed neuropsychological assessment of our study was to rule out at baseline and follow-up even mild cognitive alterations. Subjects were pre-screened by MMSE which had to be higher than 24/30 to be submitted to the screening phase; even though this MMSE has been shown elsewhere to be too low to correctly detect cognitively intact subjects (e.g. O'Bryant et al., 2008), it should be noted that a) all of these subjects after MMSE underwent an extensive neuropsychological test battery which had to be within normal values for all of them, both at T0 and at T1, to be included in the analysis; b) none of them were complaining for any subjective cognitive alteration, nor any cognitive alteration was reported by their caregivers/partners both at T0 and follow-up; d) MMSE values did not change significantly, and was never below 24, at follow up, too. Finally, LC parameters did not correlate with MMSE score.

As per the study design, subjects analyzed in our study did not show any cognitive impairment also at follow-up and there was only a slight variability among subjects for all cognitive tests. Interestingly, our cohort presented a lower mean educational level (9.43 + 4.42 years, which is in line with that of the general Italian population aged 60–80 years) compared to subjects studied by other authors (e.g. Betts et al., 2017 – mean educational level 16.0 + 2.0 years). Since it has been observed that LC assessed by MRI may correlate with cognitive reserve (Clewett et al., 2016), such a feature may partly contribute to explain why in the present study it could not observe any relation between LC and neuropsychological performance.

The age range assessed in the present study is similar to the one selected in clinical trials on neurodegenerative disorders. Setting up an appropriate control group formed by cognitively healthy subjects forms the basis for the study in age-matched patients with neurodegenerative disease, in which LC may play a key pathogenic role.

This study has some main limitations. It has been already mentioned above the reason why this age range (i.e. 60–80 years) was selected; however, we are aware that excluding subjects older than 80 years might have limited the power of our analysis, and thus a possible correlation between LC- and age occurring in older people might be missed. Moreover, the present analysis might have been affected also by the relatively low number of subjects included, and, as already discussed, the native space approach did not allow for regional analysis. Thus, potential age-related alterations of different LC sub-regions could not be assessed by the present analysis, and this constitutes a further limitation of the study. Finally, it is worth mentioning that the interpretation of our results is based on the hypothesis that LC contrast depends largely on neuromelanin content within the LC itself, in line with what claimed also by others (e.g. Clewett et al., 2016; Betts et al., 2017, 2019a, b; Liu et al., 2019, 2020). However, some authors have hypothesized that different mechanisms might contribute to LC contrast as well, including the magnetization transfer effect (Keren et al., 2015; Priovoulos et al., 2018). This should also be kept into account in the interpretation of the present data.

## Conclusions

In conclusion, this study did not show any significant age-related differences in MRI-estimated number of LC Voxels and LC-CR in subjects aged 60–80 years who maintained normal cognition over a one-year follow-up, These findings would suggest that subjects with these characteristics could be used as reference control group for future studies on LC-MRI in patients with neurodegenerative disorders.

## Supplementary Information

Supplementary Table 1 (DOC 33 kb)

Supplementary Figure 1 Correlation between the LC parameters Vox and LC-CR. Distribution of volumetric parameter Vox (Y-axis) and intensity parameter LC-CR (x-axis) in the subjects included in the study. No significant correlation between Vox and LC-CR was detected. r: coefficient of Pearson. (PNG 502 kb)

High Resolution Image (TIF 98.5 KB)

Supplementary Figure 2 Correlation between the LC parameters and MMSE scores at baseline (T0) and at the end of the follow-up period (T1). Distribution of LC parameters (Y-axis) LC-CR (graphs **A** and **C**), and Vox (graphs **B** and **D**), with MMSE scores of the subjects included in the study, at baseline (T0, graphs **A** and **B)** and T1 (graphs **C** and **D**). No significant correlation between Vox or LC-CR with MMSE scores was found, neither at baseline nor at the end of the 1 year follow-up. r: coefficient of Pearson. (PNG 0.98 mb)

High Resolution Image (TIF 164 KB)

## Data Availability

The data that support the findings of this study are available from the corresponding author upon reasonable request.

## References

[CR1] Aston-Jones G, Cohen JD (2005). An integrative theory of locus Coeruleus-norepinephrine function: Adaptive gain and optimal performance. Annual Review of Neuroscience.

[CR2] Berridge CW, Waterhouse BD (2003). The locus coeruleus–noradrenergic system: Modulation of behavioral state and state-dependent cognitive processes. Brain Research Reviews.

[CR3] Berridge, C. W., Arnsten, A. F. T., & Foote, S. L. (1993). Noradrenergic modulation of cognitive function: Clinical implications of anatomical, electrophysiological and behavioural studies in animal models. *Psychological Medicine*. 10.1017/S0033291700025332 10.1017/s0033291700025332 8234565

[CR4] Betts MJ, Cardenas-Blanco A, Kanowski M, Jessen F, Düzel E (2017). In vivo MRI assessment of the human locus coeruleus along its rostrocaudal extent in young and older adults. NeuroImage.

[CR5] Betts, M. J., Cardenas-Blanco, A., Kanowski, M., Spottke, A., Teipel, S. J., Kilimann, I. et al. (2019a). Locus coeruleus MRI contrast is reduced in Alzheimer’s disease dementia and correlates with CSF Aβ levels. *Alzheimer’s and Dementia: Diagnosis, Assessment and Disease Monitoring*. 10.1016/j.dadm.2019.02.001. 10.1016/j.dadm.2019.02.001 PMC6439222 30976648

[CR6] Betts MJ, Kirilina E, Otaduy MCG, Ivanov D, Acosta-Cabronero J, Callaghan MF (2019). Locus coeruleus imaging as a biomarker for noradrenergic dysfunction in neurodegenerative diseases. Brain.

[CR7] Braak H, Del Tredici K, Rüb U, De Vos RAI, Jansen Steur ENH, Braak E (2003). Staging of brain pathology related to sporadic Parkinson’s disease. Neurobiology of Aging.

[CR8] Braak H, Thal DR, Ghebremedhin E, Del Tredici K (2011). Stages of the pathologic process in Alzheimer disease: Age categories from 1 to 100 years. Journal of Neuropathology & Experimental Neurology.

[CR9] Caffarra P, Vezzadini G, Dieci F, Zonato F, Venneri A (2002). Una versione abbreviata del test di Stroop: dati normativi nella popolazione italiana. Nuova Rivista di Neurologia.

[CR10] Carlesimo GA, Caltagirone C, Gainotti G (1996). The mental deterioration battery: Normative data, diagnostic reliability and qualitative analyses of cognitive impairment. The Group for the Standardization of the mental deterioration battery. European Neurology.

[CR11] Carlesimo G, Buccione I, Fadda L, Graceffa A, Mauri M, Lorusso S (2002). Standardizzazione di due test di memoria per uso clinico: breve racconto e figura di Rey. Nuova Rivista di Neurologia.

[CR12] Chen X, Huddleston DE, Langley J, Ahn S, Barnum CJ, Factor SA (2014). Simultaneous imaging of locus coeruleus and substantia nigra with a quantitative neuromelanin MRI approach. Magnetic Resonance Imaging.

[CR13] Clewett DV, Lee T-H, Greening S, Ponzio A, Margalit E, Mather M (2016). Neuromelanin marks the spot: Identifying a locus coeruleus biomarker of cognitive reserve in healthy aging. Neurobiology of Aging.

[CR14] Dahl MJ, Mather M, Düzel S, Bodammer NC, Lindenberger U, Kühn S, Werkle-Bergner M (2019). Rostral locus coeruleus integrity is associated with better memory performance in older adults. Nature Human Behaviour.

[CR15] Fazekas F, Chawluk JB, Alavi A, Hurtig HI, Zimmerman RA (1987). MR signal abnormalities at 1.5 T in Alzheimer’s dementia and normal aging. American Journal of Roentgenology.

[CR16] Fernandes P, Regala J, Correia F, Gonçalves-Ferreira AJ (2012). The human locus coeruleus 3-D stereotactic anatomy. Surgical and Radiologic Anatomy.

[CR17] García-Lorenzo D, Longo-Dos Santos C, Ewenczyk C, Leu-Semenescu S, Gallea C, Quattrocchi G (2013). The coeruleus/subcoeruleus complex in rapid eye movement sleep behaviour disorders in Parkinson’s disease. Brain : A Journal of Neurology.

[CR18] German DC, Walker BS, Manaye K, Smith WK, Woodward DJ, North AJ (1988). The human locus coeruleus: Computer reconstruction of cellular distribution. Journal of Neuroscience.

[CR19] Gesi M, Soldani P, Giorgi FS, Santinami A, Bonaccorsi I, Fornai F (2000). The role of the locus coeruleus in the development of Parkinson’s disease. Neuroscience and Biobehavioral Reviews.

[CR20] Giovagnoli AR, Del Pesce M, Mascheroni S, Simoncelli M, Laiacona M, Capitani E (1996). Trail making test: Normative values from 287 normal adult controls. Italian Journal of Neurological Sciences.

[CR21] Guidance for industry: bioanalytical method validation. (2001). http://www.fda.gov/downloads/Drugs/GuidanceCompliance/RegulatoryInformation/Guidances/UCM070107.pdf. Accessed June 2013.

[CR22] Jack CR, Bennett DA, Blennow K, Carrillo MC, Dunn B, Haeberlein SB (2018). NIA-AA research framework: Toward a biological definition of Alzheimer’s disease. Alzheimer’s & Dementia : The Journal of the Alzheimer’s Association.

[CR23] Kelly SC, He B, Perez SE, Ginsberg SD, Mufson EJ, Counts SE (2017). Locus coeruleus cellular and molecular pathology during the progression of Alzheimer’s disease. Acta Neuropathologica Communications.

[CR24] Keren NI, Taheri S, Vazey EM, Morgan PS, Granholm ACE, Aston-Jones GS, Eckert MA (2015). Histologic validation of locus coeruleus MRI contrast in post-mortem tissue. NeuroImage.

[CR25] Lang, M., Michelotti, C., & Bardelli, E. (2013). *WAIS-IV: Weschsler Adult Intelligence Scale IV, lettura dei risultati e intepretazione clinica*. RC Editore Ed.

[CR26] Liu, K. Y., Marijatta, F., Hämmerer, D., Acosta-Cabronero, J., Düzel, E., & Howard, R. J. (2017). Magnetic resonance imaging of the human locus coeruleus: A systematic review. *Neuroscience and Biobehavioral Reviews*. Elsevier Ltd. 10.1016/j.neubiorev.2017.10.023. 10.1016/j.neubiorev.2017.10.023 29107830

[CR27] Liu KY, Acosta-Cabronero J, Cardenas-Blanco A, Loane C, Berry AJ, Betts MJ (2019). In vivo visualization of age-related differences in the locus coeruleus. Neurobiology of Aging.

[CR28] Liu KY, Kievit RA, Tsvetanov KA, Betts MJ, Düzel E, Rowe JB (2020). Noradrenergic-dependent functions are associated with age-related locus coeruleus signal intensity differences. Nature Communications.

[CR29] Magni E, Binetti G, Bianchetti A, Rozzini R, Trabucchi M (1996). Mini-mental state examination: A normative study in Italian elderly population. European Journal of Neurology.

[CR30] Manaye KF, McIntire DD, Mann DMA, German DC (1995). Locus coeruleus cell loss in the aging human brain: A non-random process. Journal of Comparative Neurology.

[CR31] Martin-Bastida, A., Pietracupa, S., & Piccini, P. (2017, December). Neuromelanin in parkinsonian disorders: an update. *International Journal of Neuroscience. Taylor and Francis Ltd.*10.1080/00207454.2017.1325883 10.1080/00207454.2017.1325883 28460588

[CR32] Mouton PR, Pakkenberg B, Gundersen HJG, Price DL (1994). Absolute number and size of pigmented locus coeruleus neurons in young and aged individuals. Journal of Chemical Neuroanatomy.

[CR33] Nakane T, Nihashi T, Kawai H, Naganawa S (2008). Visualization of neuromelanin in the substantia nigra and locus ceruleus at 1.5T using a 3D-gradient echo sequence with magnetization transfer contrast. Magnetic Resonance in Medical Sciences.

[CR34] O'Bryant SE, Humphreys JD, Smith GE, Ivnik RJ, Graff-Radford NR, Petersen RC, Lucas JA (2008). Detecting dementia with the mini-mental state examination in highly educated individuals. Archives of Neurology.

[CR35] Ohm TG, Busch C, Bohl J (1997). Unbiased estimation of neuronal numbers in the human nucleus coeruleus during aging. Neurobiology of Aging.

[CR36] Orsini A, Grossi D, Capitani E, Laiacona M, Papagno C, Vallar G (1987). Verbal and spatial immediate memory span: Normative data from 1355 adults and 1112 children. Italian Journal of Neurological Sciences.

[CR37] Paxinos, G., & Mai, J. K. (2003). *The Human Nervous System: Second Edition*. *The Human Nervous System: Second Edition*. Elsevier Inc. 10.1016/B978-0-12-547626-3.X5000-5.

[CR38] Priovoulos N, Jacobs HIL, Ivanov D, Uludağ K, Verhey FRJ, Poser BA (2018). High-resolution in vivo imaging of human locus coeruleus by magnetization transfer MRI at 3T and 7T. NeuroImage.

[CR39] Royall DR, Cordes JA, Polk M (1998). CLOX: An executive clock drawing task. Journal of Neurology, Neurosurgery, and Psychiatry.

[CR40] Sara SJ (2009). The locus coeruleus and noradrenergic modulation of cognition. Nature Reviews Neuroscience.

[CR41] Sarazin M, Berr C, De Rotrou J, Fabrigoule C, Pasquier F, Legrain S (2007). Amnestic syndrome of the medial temporal type identifies prodromal AD: A longitudinal study. Neurology.

[CR42] Sasaki M, Shibata E, Tohyama K, Takahashi J, Otsuka K, Tsuchiya K (2006). Neuromelanin magnetic resonance imaging of locus ceruleus and substantia nigra in Parkinson’s disease. Neuroreport.

[CR43] Schwarz ST, Xing Y, Tomar P, Bajaj N, Auer DP (2017). In vivo assessment of brainstem depigmentation in Parkinson disease: Potential as a severity marker for multicenter studies. Radiology.

[CR44] Shibata E, Sasaki M, Tohyama K, Kanbara Y, Otsuka K, Ehara S, Sakai A (2006). Age-related changes in locus ceruleus on neuromelanin magnetic resonance imaging at 3 tesla. Magnetic Resonance in Medical Sciences.

[CR45] Shrout PE, Fleiss JL (1979). Intraclass correlations: Uses in assessing rater reliability. Psychological Bulletin.

[CR46] Spinnler, H., & Tognoni, G. (1987). *Standardizzazione e taratura italiana di test neuropsicologici*. MI Periodici Ed.

[CR47] Theofilas P, Ehrenberg AJ, Dunlop S, Di Lorenzo Alho AT, Nguy A, Leite REP (2017). Locus coeruleus volume and cell population changes during Alzheimer’s disease progression: A stereological study in human postmortem brains with potential implication for early-stage biomarker discovery. Alzheimers Dement.

[CR48] Zecca L, Stroppolo A, Gatti A, Tampellini D, Toscani M, Gallorini M (2004). The role of iron and copper molecules in the neuronal vulnerability of locus coeruleus and substantia nigra during aging. Proceedings of the National Academy of Sciences of the United States of America.

