# Supplementary material for: Locus Coeruleus magnetic resonance imaging in cognitively intact elderly subjects
Source: Brain Imaging Behav. 2021 Nov 5;16(3):1077–87. doi: 10.1007/s11682-021-00562-0 (PMC9107398; doi:10.1007/s11682-021-00562-0)
Supplement: Supplementary file 1 — (DOC 33 kb) [file 11682_2021_562_MOESM1_ESM.doc]

**Supplementary Table I. Inter and intra-observer analysis: intraclass correlation coefficient and coefficient of variation.**

| **LC parameters** | **ICC**  **Interobserver** | **p value**  **Interobserver** | **ICC**  **Intraobserver** | **p value**  **Intraobserver** | **CV**  **Interobserver** | **CV**  **Intraobserver** |
| --- | --- | --- | --- | --- | --- | --- |
| VOX | 0.655  (0.419-0.806) | <0.001 | 0.716  (0.508-0.836) | <0.001 | 13.9% | 10.9% |
| LC-CR | 0.947  (0.909-0.970) | <0.001 | 0.960  (0.931-0.977) | <0.001 | 0.45% | 0.39% |

**Legend:** **ICC** =Interclass Correlation Coefficient; **CV**= Coefficient of Variation
