# Supplementary figures and images for: Locus Coeruleus magnetic resonance imaging in cognitively intact elderly subjects
Source: Brain Imaging Behav. 2021 Nov 5;16(3):1077–87. doi: 10.1007/s11682-021-00562-0 (PMC9107398; doi:10.1007/s11682-021-00562-0)

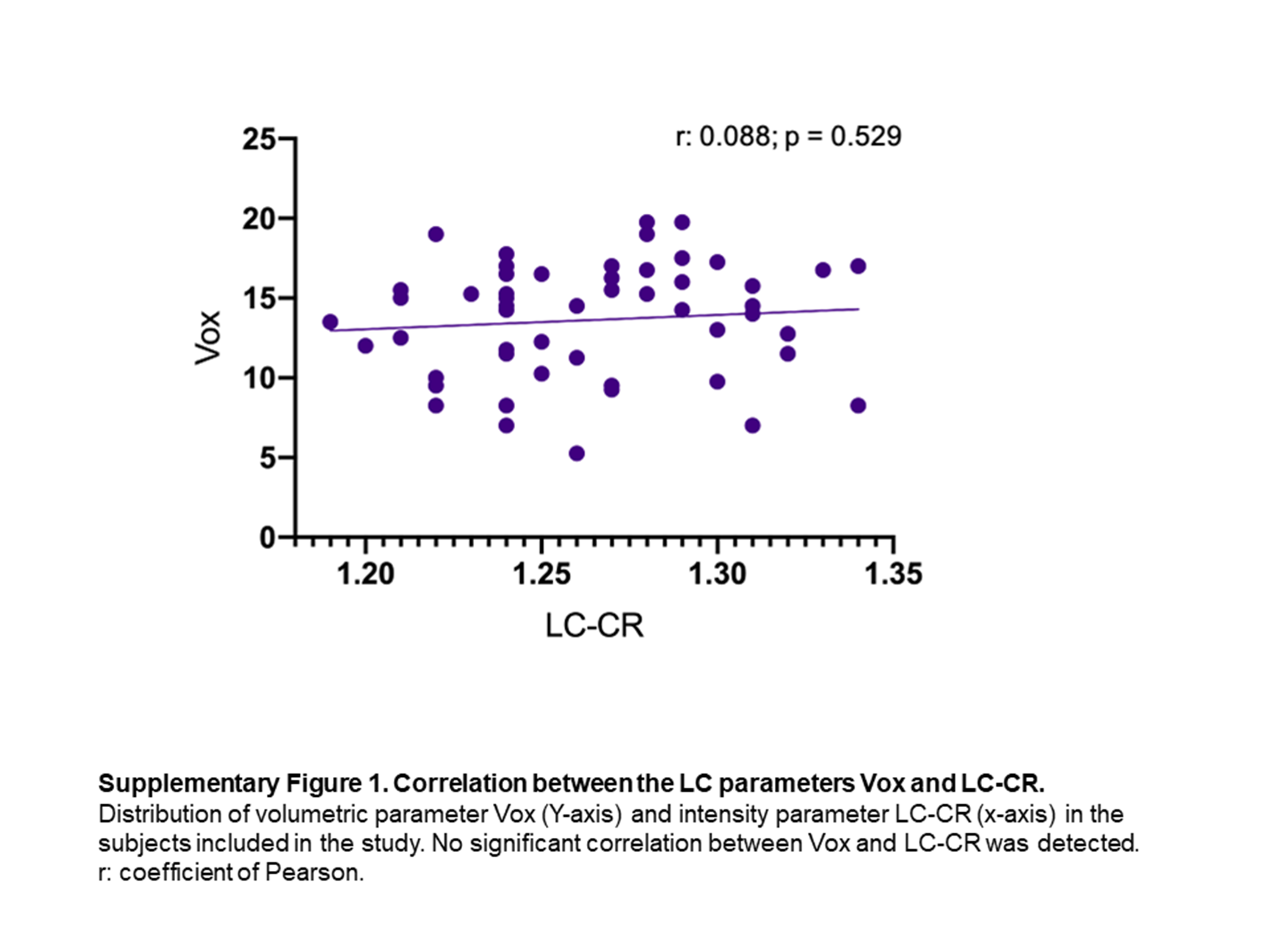

Supplement: Supplementary file 2 — Correlation between the LC parameters Vox and LC-CR. Distribution of volumetric parameter Vox (Y-axis) and intensity parameter LC-CR (x-axis) in the subjects included in the study. No significant correlation between Vox and LC-CR was detected. r: coefficient of Pearson. (PNG 502 kb) [file 11682_2021_562_Fig3_ESM.png]

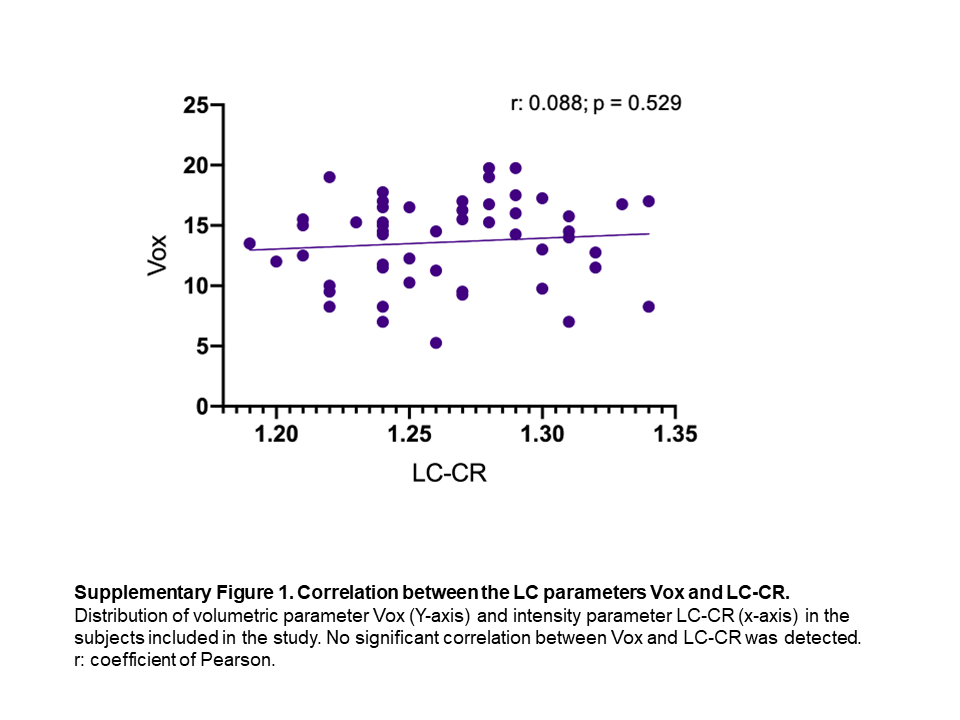

Supplement: Supplementary file 3 — High Resolution Image (TIF 98.5 KB) [file 11682_2021_562_MOESM2_ESM.tif]

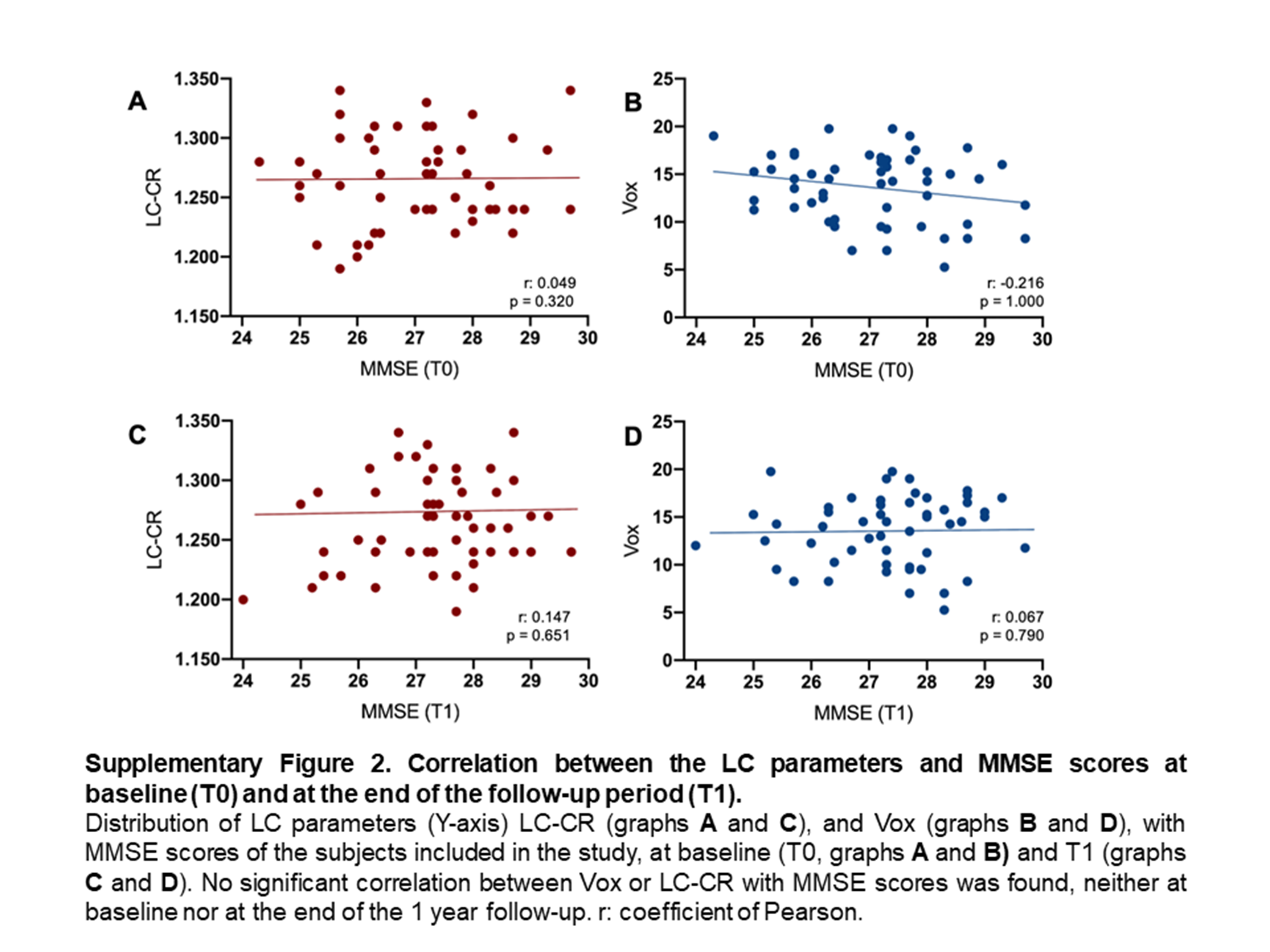

Supplement: Supplementary file 4 — Correlation between the LC parameters and MMSE scores at baseline (T0) and at the end of the follow-up period (T1). Distribution of LC parameters (Y-axis) LC-CR (graphs A and C), and Vox (graphs B and D), with MMSE scores of the subjects included in the study, at baseline (T0, graphs A and B) and T1 (graphs C and D). No significant correlation between Vox or LC-CR with MMSE scores was found, neither at baseline nor at the end of the 1 year follow-up. r: coefficient of Pearson. (PNG 0.98 mb) [file 11682_2021_562_Fig4_ESM.png]

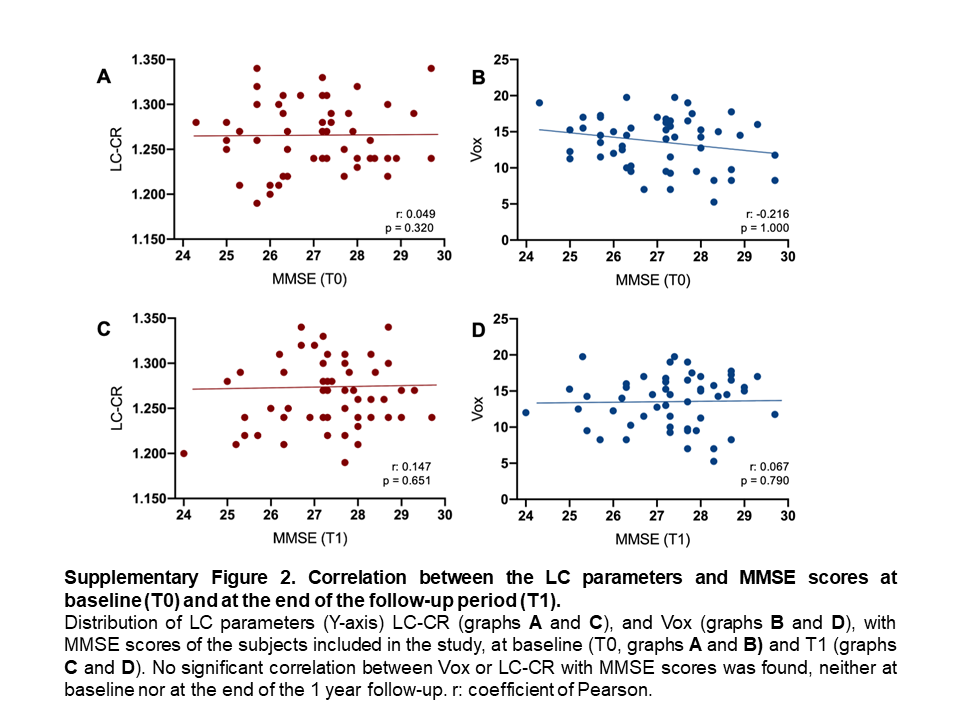

Supplement: Supplementary file 5 — High Resolution Image (TIF 164 KB) [file 11682_2021_562_MOESM3_ESM.tif]
